# MiR-23b-3p reduces the proliferation, migration and invasion of cervical cancer cell lines via the reduction of c-Met expression

**DOI:** 10.1038/s41598-020-60143-x

**Published:** 2020-02-24

**Authors:** Gabriela Elizabeth Campos-Viguri, Oscar Peralta-Zaragoza, Hilda Jiménez-Wences, Alma Edith Longinos-González, Carlos Alberto Castañón-Sánchez, Miriam Ramírez-Carrillo, César López Camarillo, Eduardo Castañeda-Saucedo, Marco Antonio Jiménez-López, Dinorah Nashely Martínez-Carrillo, Gloria Fernández-Tilapa

**Affiliations:** 1 Laboratorio de Investigación Clínica. Facultad de Ciencias Químico-Biológicas, Universidad Autónoma de Guerrero. Av. Lázaro Cárdenas S/N, Colonia Haciendita, Chilpancingo, 39070 Guerrero México; 2 Dirección de Infecciones Crónicas y Cáncer; Centro de Investigación en Enfermedades Infecciosas. Instituto Nacional de Salud Pública. Av. Universidad No. 655, Cerrada los Pinos y Caminera. Colonia Santa María Ahuacatitlán, Cuernavaca, 62100 Morelos México; 3 Hospital Regional de Alta Especialidad de Oaxaca. Av. Aldama S/N, Colonia Centro, San Bartolo Coyotepec, 71256 Oaxaca México; 4 Laboratorio de Química de Productos Naturales, Facultad de Farmacia, Universidad Autónoma del Estado de Morelos. Av. Universidad No. 1001, Colonia Chamilpa, Cuernavaca, 62209 Morelos México; 5 Posgrado en Ciencias Genómicas, Universidad Autónoma de la Ciudad de México, Ciudad de México, México; 6 Laboratorio de Biología Celular del Cáncer. Facultad de Ciencias Químico-Biológicas, Universidad Autónoma de Guerrero. Av. Lázaro Cárdenas S/N, Colonia Haciendita, Chilpancingo, 39070 Guerrero México; 7 Instituto Estatal de Cancerología “Dr. Arturo Beltrán Ortega”. Av. Adolfo Ruiz Cortines, No. 128-A. Colonia Alta Progreso, Acapulco de Juárez, 39570 Guerrero México

**Keywords:** Cervical cancer, Cervical cancer

## Abstract

Malignant transformation and progression in cancer is associated with the altered expression of multiple miRNAs, which are considered as post-transcriptional regulators of genes participating in various cellular processes. Although, it has been proposed that miR-23b-3p acts as a tumor suppressor in cervical cancer (CC), not all the pathways through which it alters the cellular processes have been described. The present study examines whether miR-23b-3p directly represses the c-Met expression and that consequently modifies the proliferation, migration and invasion of C33A and CaSki cells. c-Met has five microRNA response elements (MREs) for miR-23b-3p in the 3′-UTR region. The ectopic overexpression of miR-23b-3p significantly reduces c-Met expression in C33A and CaSki cells. The overexpression of miR-23b-3p reduces proliferation, migration and invasion of CaSki cells and the proliferation and invasion in C33A cells. In CaSki cells, the activation of Gab1 and Fak, downstream of c-Met, is reduced in response to the overexpression of miR-23b-3p. Together, the results in the present study indicate that miR-23b-3p is a tumor suppressor that modulates the progression of CC via post-transcriptional regulation of the c-Met oncogene.

## Introduction

Cervical cancer (CC) is the fourth most common malignancy in women around the world, with 569,847 newly diagnosed cases and 311,365 deaths registered in 2018^1^. The late detection of CC increases the risk of metastasis and death, with a third of CC patients diagnosed in the advanced stages of the disease^2^. This has driven the search for progression and prognosis markers which enable the improvement of treatment scheme selection.

While persistent high-risk human papillomavirus (HR HPV) infection plays a central role in the genesis of CC^3^, other factors also contribute to the origin and progression of this malignancy. Some mutations, modifications in specific and global DNA methylation, changes to the expression profiles of chromatin-modifying enzymes and the aberrant expression of microRNAs (miRNAs) are events frequently detected in CC^4,5^. Regulating gene expression at a post-transcriptional level, miRNAs participate in controlling proliferation, metabolism, differentiation and apoptosis^6–8^. These are non-coding RNAs comprising 19–25 nucleotides, which induce the degradation of the target mRNA or repress its translation into protein via its total or partial binding to complementary sequences situated in the 3′-UTR region of the transcript^6^.

Although the malignant transformation and progression of cancer are associated with the altered expression of multiple miRNAs, it is not known whether this alteration marks the beginning of cellular transformation or is a consequence of it. Based on its level of expression and the biological function of the genes that they regulate; miRNAs are classified into oncomiRs and tumor suppressor miRNAs^7^. OncomiRs regulate tumor suppressor genes and increase their expression in cancerous tissue, while tumor suppressor miRNAs regulate oncogenes and reduce their expression in the tumor^7^. The characterization of miRNA profiles in specific tumors may be useful for early diagnosis, the estimation of both progression and prognosis, and the choice of treatment strategies.

The expression of miR-23b-3p is reduced in a group of malignant tumors^9–11^, including CC^12^. In CC cell lines, the level of p53 and the expression of miR-23b-3p increase in response to the silencing of the E6 oncoprotein of HPV16^13^. In a prior study, our research group found that the level of miR-23b-3p expression is reduced in CC tissues and cell lines, a reduction that correlates to changes in the methylation of the promoter of this miRNA^12^.

Although few target genes of miR-23b-3p have been characterized and only the participation of this miRNA in the regulation of some cellular processes has been documented, miR-23b-3p has been proposed as a tumor suppressor in CC, with important functions in stages prior to the metastasis of the cancer^10,11,14^. The results of functional analysis suggest that the ectopic expression of miR-23b-3p represses cell growth, migration, and invasion, as well as tumor angiogenesis, through the regulation of the oncogenes FZD70, MAP3K1, PAK2, TGFβR2, RRAS2 and uPA in colon cancer^10^. In ovarian cancer, the overexpression of miR-23b-3p inhibits proliferation and carcinogenesis via RUNX2^11^. In the tissue of patients with bladder cancer^14^ or oral squamous cell carcinoma^15^, the endogenous expression of miR-23b-3p is significantly reduced. In the cell lines of both types of cancer, the restoration of miR-23b-3p via ectopic expression significantly reduces cellular migration and invasion, while miR-23b-3p has been confirmed to directly regulate the expression of the receptor tyrosine kinase c-Met in both cases^14,15^.

In normal conditions, c-Met participates in the regulation of embryonic development, tissue repair and cell regeneration. In CC^16^ and other types of cancer, the increased expression and activation of c-Met promotes cellular proliferation, survival, migration and invasion. In these malignancies, c-Met is known as an oncogene^14,15,17–19^. In the CC cell line SiHa, the repression of c-Met by miR-23b-3p promotes apoptosis^20^, although it is not known whether this miRNA affects other cellular processes associated with CC. The generation of new knowledge on molecular pathways regulated by miR-23b-3p will improve the understanding of the mechanisms that contribute to the alteration of cellular processes that participate in the oncogenesis and metastasis of CC.

In the present study, the CC cell lines C33A and CaSki were used to ascertain whether miR-23b-3p directly represses the expression of c-Met and whether this repression modifies the proliferation, migration and invasion of cells. Additional objectives of the present research were to evaluate the activation of Gab1 and Fak in response to the ectopic expression of miR-23b-3p and analyze the level of c-Met mRNA and protein in HPV16-positive CC tissues. Together, the results of the present study show that c-Met is a target gene of miR-23b-3p and that the ectopic overexpression of this miRNA reduces the proliferation, migration and invasion of C33A and CaSki cells. In CaSki cells, the ectopic overexpression of miR-23b-3p significantly reduces the activation of Gab1 and Fak. In the tissue of HPV16-positive CC patients, the expression of c-Met is heterogenous.

## Results

### The expression of miR-23b-3p is differentially restored in CC cell lines

**Figure 1:**
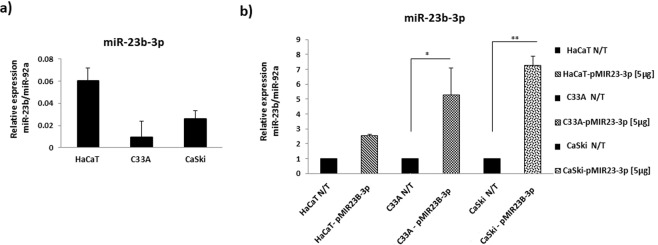
miR-23b-3p is reduced in CC cell lines. **(a)** The expression of miR-23b-3p in C33A and CaSki cells is lower than in HaCaT cells. **(b)** Transfection with the plasmid pMIR23B-3p significantly increases the expression of miR-23b-3p in CC cell lines. The levels of miR-23b-3p expression were analyzed via the 2^−ΔCt^ method and normalized to miR-92a. Five micrograms of the plasmid pMIR23B-3p was used for the assays. N/T: Non-transfected. The data are expressed in averages + SE. * p < 0.05, **p < 0.01.

The level of expression of miR-23b-3p in CC cell lines was compared with that found in HaCaT cells, with C33A and CaSki cells found to express lower levels of miR-23b-3p than HaCaT cells (Fig. 1a).

In order to overexpress miR-23b-3p in CC and HaCaT cells, the plasmid pMIR23B-3p was generated and the efficiency of the transfection in the three cell lines was tested, with pMIR23b-3p significantly increasing the expression of miR-23b-3p in C33A and CaSki cells (Fig. 1b).

### The overexpression of miR-23b-3p reduces proliferation, migration and invasion in CC cell lines

**Figure 2:**
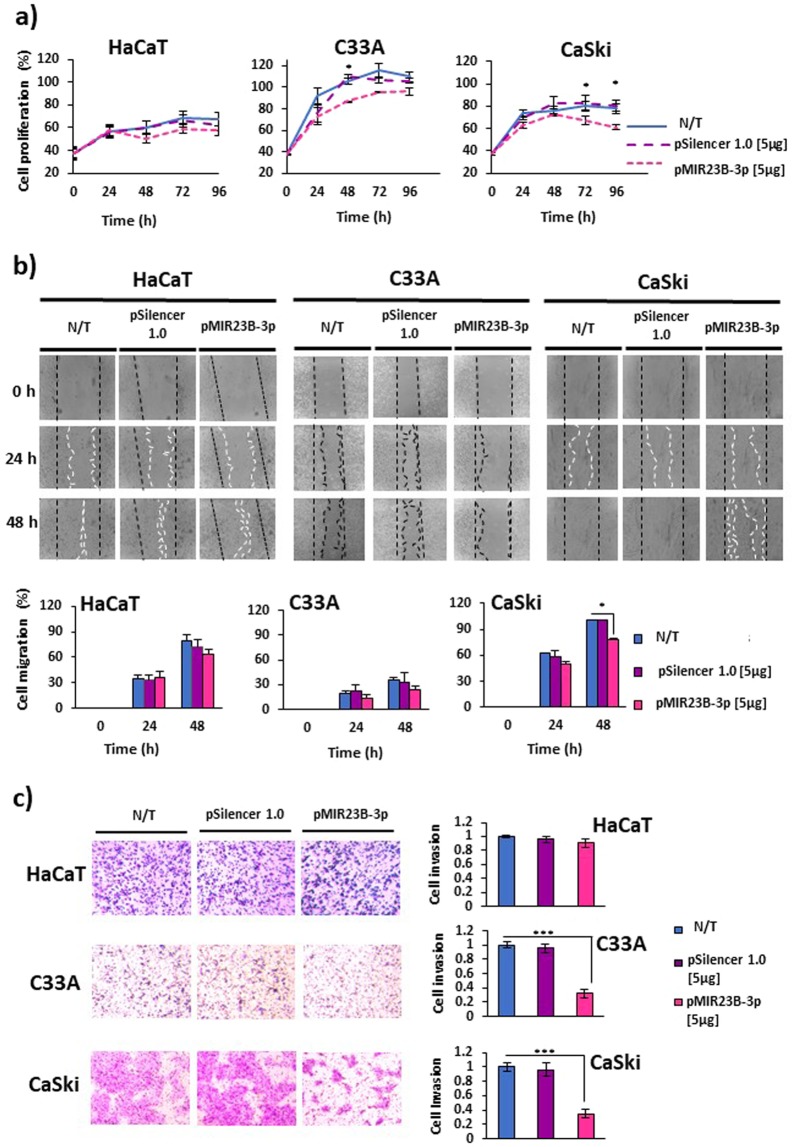
Effect of miR-23b-3p on the proliferation, migration and invasion of C33A and CaSki cells. **(a)** The proliferation of CC and HaCaT cells was evaluated via MTS assays. The proliferation of C33A and CaSki cells was reduced at 48 and 72 h, respectively, after transfection with the plasmid pMIR23B-3p. **(b)** Cellular migration was evaluated via wound healing assay. The images were captured at 0, 24 and 48 h after the stria was made. The migration of CaSki cells significantly reduced at 48 h after transfection with the plasmid pMIR23B-3p. **(c)** Cellular invasion was measured via Transwell assays. In the microphotographs of the C33A cells transfected with the plasmid pMIR23B-3p, crystal violet residues predominate, while the number of invader cells is very low. The overexpression of miR-23b-3p significantly reduced the invasion of C33A and CaSki cells. Five micrograms of the plasmid pMIR23B-3p and pSilencer 1.0-U6 (without DNA insert) were used in each transfection assay. N/T: non-transfected. The data are expressed in averages + SE. *p < 0.05, ***p < 0.001 compared to the negative control.

Functional assays were carried out in order to determine the effect of miR-23b-3p on the proliferation, migration and invasion of the CC cells. MTS assays were conducted at 0, 24, 48, 72 and 96 hours after transfection with the plasmid pMIR23B-3p. The results revealed that miR-23b-3p reduces cellular proliferation. At 48 h post-transfection, the proliferation of C33A was reduced by approximately 20% more than that of the N/T cells, while the proliferation of CaSki cells fell by 15% at 72 h and by 20% at 96 h post-transfection (Fig. 2a).

Given that mobility is a key process in the metastasis of cancer, the present study evaluated the effect of miR-23b-3p on cellular migration via wound-healing assays. The results show that, 48 h after transfection, the overexpression of miR-23b-3p significantly reduces the migration of CaSki cells (Fig. 2b). In order to verify whether the overexpression of miR-23b-3p affects cellular invasion, Transwell invasion assays were undertaken with matrigel simulating the extracellular matrix (ECM). The number of C33A and CaSki invader cells was significantly lower in those cells with the overexpression of miR-23b-3p than in either those cells transfected with the plasmid pSilencer 1.0-U6 or N/T cells (Fig. 2c). The microphotographs suggest, in CC cells, invasion patterns and differential migratory phenotypes. The C33A cells seem to invade the matrigel individually and present amoeboid migration. This type of mobility is characteristic of cells which are incapable of lysing the ECM and move through spaces surrounding the ECM^21^. The CaSki cells invade the matrigel collectively and present mesenchymal migration, which is reflected in a greater number of invader cells.

Together, these results show that miR-23b-3p reduces the proliferation, migration and invasion of CaSki cells and the proliferation and invasion of C33A cells.

### c-Met is a direct target of miR-23b-3p in CC cells

**Figure 3:**
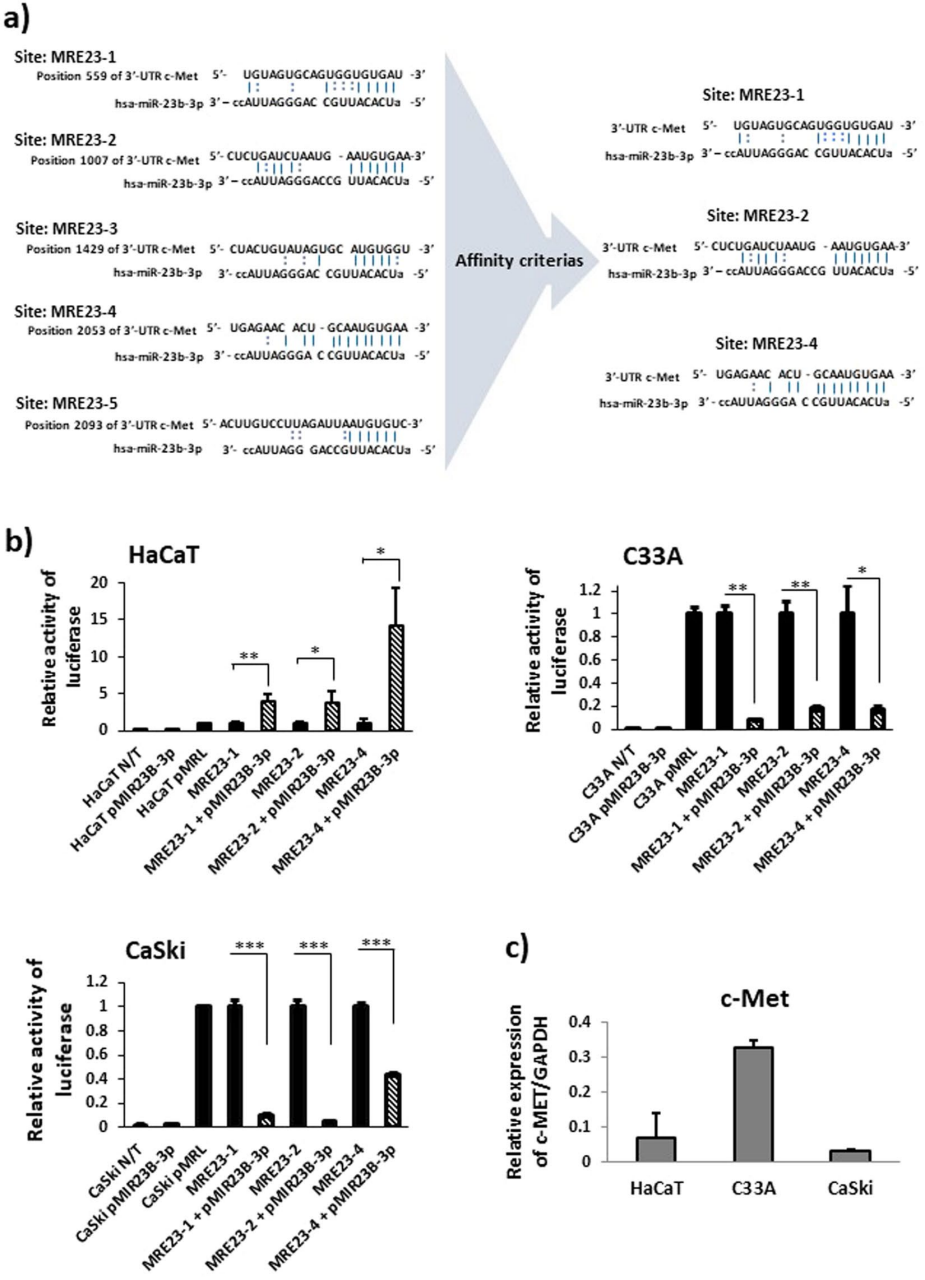
miR-23b-3p represses c-Met in C33A and CaSki cells through its interaction with three MRE sites from the 3′-UTR region of c-Met. **(a)** The MRE23-1, MRE23-2 and MRE23-4 located in the 3′-UTR region of c-Met mRNA are most likely to interact with miR-23b-3p. **(b)** c-Met is a direct target of miR-23b-3p in CC cells. The binding of miR-23b-3p to the three MRE23 sites inhibits luciferase activity in C33A and CaSki cells. The luciferase relative activity was detected 48 h post-transfection. N/T: non-transfected; pMRL: pMiR-Report-Luciferase (without DNA insert). *p < 0.05, **p < 0.01, ***p < 0.001 compared to simple transfection. **(c)** The expression of c-Met was determined via RT-qPCR. The c-Met expression levels were normalized with GAPDH and analyzed via the ΔCt method. The data were expressed as averages + SE.

In order to verify the existence and location of the MREs for miR-23-3p in the 3′-UTR region of c-Met mRNA, an *in silico* analysis was conducted using the TargetScan, miRTarBase, microRNA.org, miRDB, RNA22, and PicTar algorithms. Based on affinity criteria (hybridization score and type), five MRE sites for miR-23b-3p were found in the 3′-UTR region of c-Met, while MRE23-1, MRE23-2, and MRE23-4 were found more likely to hybridize with miR-23b-3p (Fig. 3a). In order to verify whether the three MRE23 sites are recognized by miR-23b-3p in the intracellular context of HaCaT, C33A, and CaSki, the plasmids pMRE23cMETLuc1, pMRE23cMETLuc2, and pMRE23cMETLuc3, which contain the sequences MRE23-1, MRE23-2 and MRE23-4, respectively, were generated, while luciferase reporter assays were also designed. For the reporter assays, the plasmids pMRE23cMETLuc1, pMRE23cMETLuc2, and pMRE23cMETLuc3 were transfected either individually or in co-transfection with the plasmid pMIR23B-3p, in the HaCaT, C33A and CaSki cells. As predicted, miR-23b-3p hybridizes with the sites MRE23-1, 2 and 4 and significantly reduces the activity relative to the luciferase in the C33A and CaSki cells (Fig. 3b). Although miR-23b-3p hybridizes with the three MRE23 sites, the functional effect varies among sites and cell lines.

The level of c-Met expression in CC cell lines and HaCaT cells was determined, with c-Met expression found to be higher in the C33A cells (Fig. 3c).

**Figure 4:**
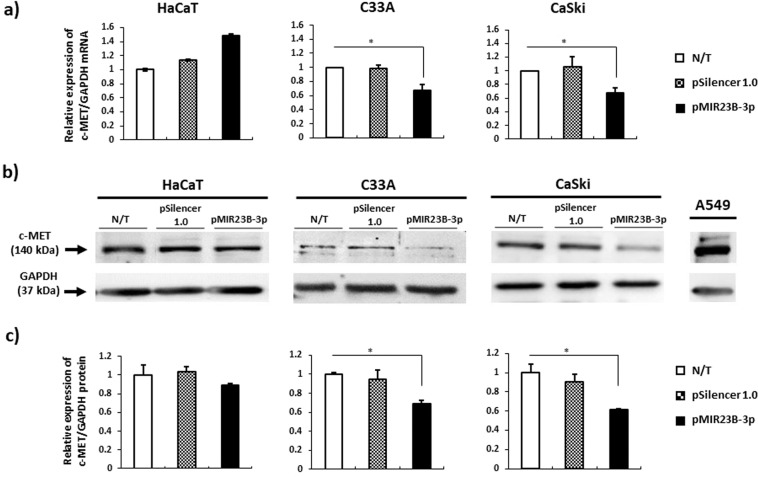
The overexpression of miR-23b-3p reduces the levels of c-Met mRNA and protein in C33A and CaSki cells. **(a)** miR-23b-3p significantly reduced the expression of c-Met mRNA. The levels of c-Met mRNA were determined via RT-qPCR and analyzed via the 2^−ΔCt^ method. **(b)** In overexpression conditions, miR-23b-3p significantly reduces c-Met protein levels. The levels of mRNA and protein were normalized using GAPDH. The cell line A549 was used as a positive control for the expression of c-Met in the Western Blot assays. **(c)** Densitometric analysis of the expression of c-Met. N/T: non-transfected. The data are expressed as averages + SE. *p < 0.05.

The RT-qPCR and Western blot assays conducted revealed that the overexpression of miR-23b-3p significantly reduced the levels of mRNA and c-Met protein in C33A and CaSki cells (Fig. 4; see Supplementary Fig. S1).

### miR-23b-3p modifies the activation of Gab1 and Fak in CaSki cells

**Figure 5:**
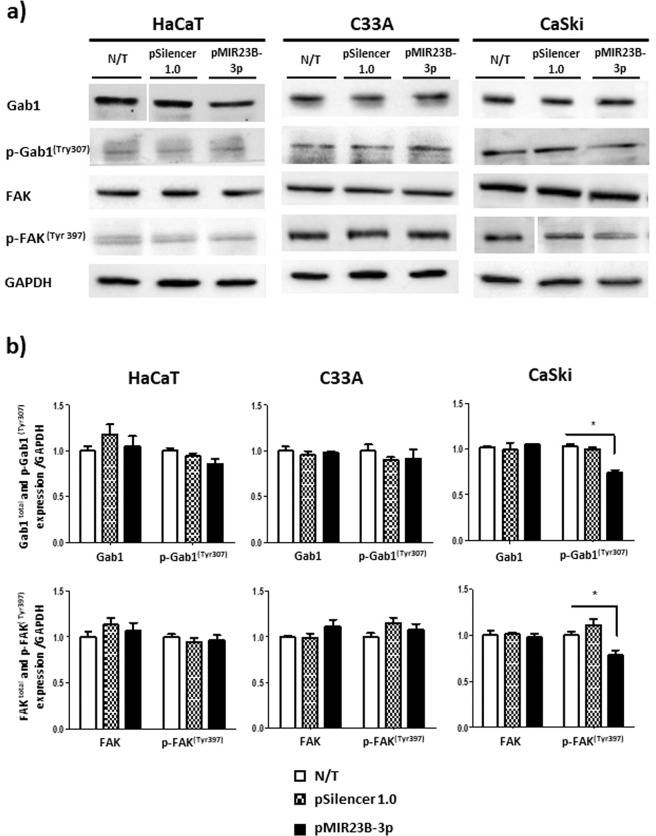
Effect of miR-23b-3p on the activation of Gab1 and Fak. **(a)** Gab1, Fak, p-Gab1(Tyr307), and p-Fak (Tyr397) levels in HaCaT, C33A and CaSki cells. **(b)** The overexpression of miR-23b-3p significantly reduces the activation of Gab1 and Fak in CaSki cells. The expression of the proteins was normalized with the GAPDH load control. N/T: non-transfected cells. The data are expressed as averages ± SE. *p < 0.05.

In order to evaluate whether the effect of miR-23b-3p on the proliferation, migration, and invasion of CC cells is related to the activation of proteins downstream of c-Met, such as the adaptor protein Gab1 and the kinase Fak, the level of p-Gab1 and p-Fak was determined. Both proteins participate in signaling pathways induced by c-Met. After transfection with the plasmid pMIR23B-3p, significant reductions in the activation of Gab1 and Fak were found solely in the CaSki cells (Fig. 5; see Supplementary Figs. S2, S3 and S4).

### c-Met is overexpressed in CC tissues

**Figure 6:**
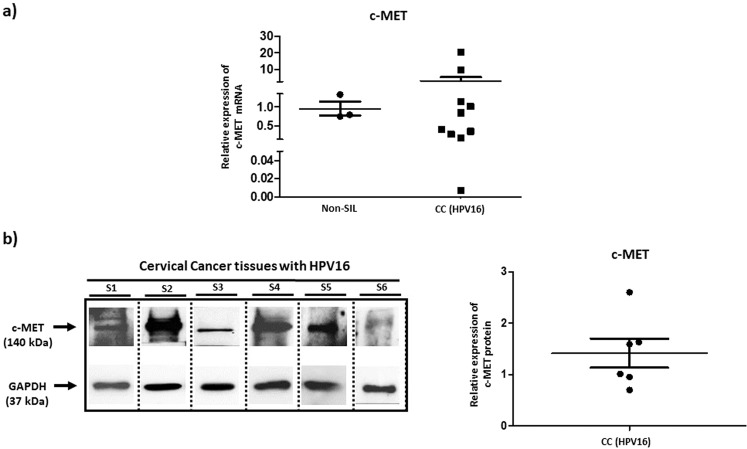
Levels of c-Met expression in CC tissue. **(a)** Level of relative c-Met expression in cervical scraping samples taken from non-SIL patients and in CC tissues, determined via RT-qPCR. The expression of c-Met mRNA is higher in CC tissues than in non-SIL scraping samples. The levels of c-Met expression were normalized with those from GAPDH and analyzed via the ΔCt method. The comparison among groups was undertaken via the Mann Whitney test. **(b)** The expression of the c-Met protein was heterogenous in CC tissues. The image from the Western Blot assay was constructed based on bands located in different positions in the same membrane and independent assays. The expression of c-Met was normalized with the expression of GAPDH protein. S: Sample.

With the aim of examining the level of c-Met expression in CC tissues, c-Met mRNA was quantified via RT-qPCR assays conducted on ten CC tissues (HPV16-positive) and three non-SIL samples. Previously, it was observed that these CC tissues present a reduced expression of miR-23b-3p. Significant differences were not found between the levels of c-Met expression in CC tissues and non-SIL samples (Fig. 6a). The expression of the c-Met protein in CC tissue was evaluated via Western Blot assays conducted on six samples taken from patients with a CC diagnosis. The results indicate that the c-Met protein is expressed heterogeneously in HPV16-positive CC patients (Fig. 6b; see Supplementary Fig. S5).

Together, the results indicate that, in CC, miR-23b-3p participates in the modulation of cellular proliferation, migration, and invasion through the regulation of the expression of c-Met and the activation of proteins downstream of c-Met. The results strengthen the hypothesis that miR-23b-3p is a tumor suppressor in CC.

## Discussion

Due to the fact that the specific functions of miR-23b-3p in CC are yet to be established, the present research explored the effect of the overexpression of miR-23b-3p on cellular proliferation, migration and invasion processes. Moreover, it sought to show that c-Met is a direct target of miR-23b-3p in CC cell lines and verify whether the overexpression of miRNA is related to the activation of Gab1 and Fak.

The most significant findings were: (1) As expected, the expression of miR-23b-3p is reduced in CC cell lines; (2) The overexpression of miR-23b-3p reduces the proliferation of C33A and CaSki cells, contributes significantly to reducing the invasion of C33A cells, and significantly reduces the migration and invasion of CaSki cells; (3) c-Met mRNA has five binding sites for miR-23b-3p in its 3′-UTR region; (4) c-Met is a direct target of miR-23b-3p in C33A and CaSki cells, but not in HaCaT cells; (5) The overexpression of miR-23b-3p significantly reduces the expression of mRNA and c-Met protein in C33A and CaSki cells; (6) In CaSki cells, the activation of Gab1 and Fak reduces in response to the overexpression of miR-23b-3p; and, (7) The expression of c-Met is heterogenous among CC tissues.

The reduced expression of miR-23b-3p in C33A and CaSki cells observed in the present study is a finding that concurs with that reported by Lui *et al*.^22^ and Li *et al*.^23^. Au-Yeung *et al*.^13^ found that, in SiHa cells, the silencing of the E6 oncoprotein of HPV16 induces the increased expression of miR-23b-3p and observed that p53 binds to the promoter region of miRNA. The present study found that the level of miR-23b-3p in HPV-negative C33A cells is lower than in HPV16-positive CaSki cells. This finding could result from the characteristics of each cell line, the tissue and the type of cancer to which they pertain (C33A: non-differentiated carcinoma *in situ*; and, CaSki: metastatic carcinoma), the differential expression of transcription factors for miR-23b-3p, and the multiple mechanisms that regulate gene expression. In HPV16-positive CC, the oncoproteins E6 and E7 modulate the expression of cellular miRNAs via different molecular mechanisms^12,24,25^. Therefore, the differences in miR-23b-3p expression between C33A and CaSki cells may also be due to the following: (1) The expression of DROSHA and DICER is higher in CaSki than in C33A cells^24^, which results in higher levels of both processing and concentration of the precursor and mature molecules of miR-23b-3p in CaSki cells; (2) p53 partially modulates the expression of miR-23b-3p via *cis* regulation^26^, and C33A expresses a muted form lacking function as a transcription factor;^27^ and, (3) The percentage of methylated copies of the miR-23b-3p promoter varies between both cell lines^12^. On the other hand, the level of miR-23b-3p expression in epithelial non-tumorous HaCaT cells was higher than in CaSki and C33A cells. This finding strengthens the hypothesis that this miRNA is a tumor suppressor in CC^12,20^, as occurs in colon cancer^10^, oral squamous cell carcinoma^15^, and bladder cancer^14,28^.

As a tumor suppressor, miR-23b-3p participates in the regulation of cellular proliferation, migration and invasion via the regulation of c-Met in solid tumors^14,15^. In the present study, the proliferation of C33A and CaSki cells reduced after 48 and 72 h, respectively, of miR-23b-3p overexpression. The reduced proliferation of C33A and CaSki found in the present study coincides with the function of c-Met (regulation of cellular survival, apoptosis and proliferation)^17^. Au-Yeung *et al*.^20^ found that the restoration of miR-23b-3p induces the apoptosis of SiHa cells after 48 h.

The overexpression of miR-23b-3p in CaSki cells significantly reduces cellular migration and invasion, while, in C33A cells, miR-23b-3p significantly reduces cellular invasion. In HeLa cells, the miR-23b/MAP1K axis modulates cellular growth and migration^23^. In colon cancer, this miRNA reduces the expression of vimentin and increases the expression of E-cadherin, favoring the generation of a stable epithelial phenotype, with reduced cellular migration and invasion^10^. Epithelial-mesenchymal transition (EMT) can occur in CC and is related to invasion and metastasis^29^. Cellular mobility, migration and invasion, as well as EMT, are regulated by signals which are induced by c-Met and activate the protein Fak^17^. The overexpression of miR-23b-3p induces a reduction in the expression of both c-Met and the signals emitted by the receptor, which partially explains the reduced migration and invasion of the C33A and CaSki cells found by the present research. In order to invade the ECM and the surrounding tissue, the tumor cells develop either a collective or individual pattern of invasion. Cancer cells are able to migrate slowly following the amoeboid phenotype, or more efficiently following the mesenchymal phenotype^30^. The findings of the present study suggest that C33A follows a pattern of individual invasion and migrates in amoeboid form, characteristics that explain the low quantity of cells that migrate to the center of the stria in the migration assay and the lower number of invader cells found in the Transwell assays. On the other hand, the higher number of invader CaSki cells may be due to collective invasion and mesenchymal migration, with this type of cells presenting greater mobility in groupings. The results of the present study suggest that miR-23b-3p modulates different types of migration and invasion in CC. However, the characterization of the invasive and migratory patterns of C33A and CaSki requires more detailed research and the determination of specific markers and mechanisms.

Our *in silico* analysis, supported by six bioinformatic algorithms, revealed that the 3′-UTR region of c-Met mRNA contains five potential MRE sites for the binding of miR-23b-3p, which is one MRE site more than those reported by Salvi *et al*.^31^, who used two bioinformatic platforms. Three of the five MRE sites of c-Met that had a greater affinity for miR-23b-3p *in silico* were evaluated via luciferase reporter assays. The MREs studied were of 6mer (MRE23-1) and 8mer (MRE23-2 and 4) type. The MRE23-1 was validated by Au-Yeung *et al*.^20^ in SiHa cells, while the three MRE23 sites selected were evaluated by Salvi *et al*.^31^ in SKHep1C3 cells derived from hepatocellular carcinoma. Salvi *et al*.^31^ found that the hybridization c-Met:miR-23b-3p reduced the activity of luciferase by 26% for the MRE23-1 site, by 20% for MRE23-2 and by 10% for MRE23-4. The present study found that, in C33A and CaSki cells, which overexpress miR-23-3p, luciferase relative activity reduced by between 50% and 95% for the three MRE23s. It is probable that the cellular context determines recognition, the stability of the hybridization and the repression of c-Met by miR-23b-3p. These results confirm that c-Met is a direct target of miR-23b-3p in C33A and CaSki cells. The binding of other endogenous miRNAs to the same MREs in c-Met mRNA may also contribute to reducing luciferase activity. The platform TargetScan indicates that miR-23a-3p, miR-23c and miR-130a-5p recognize the same MREs as miR-23b-3p. In contrast, the complex c-Met:miR-23b-3p is not formed in HaCaT cells, while, surprisingly, luciferase activity increased in the co-transfection assays, especially in the cells co-transfected with the plasmids pMRE23cMETLuc3 (MRE23-4) and pMIR23B-3p. It is possible that the activity of the CMV promoter in the expression plasmid used varies depending on the cellular context^32,33^ and its own regulation mechanisms^34^. In HaCaT cells, the protein Argonaut 2 (AGO2) is repressed by miRNAs and, consequently, reduces the formation and efficiency of the miRISC complex, which enables the translation of proteins regulated by miRNAs^35^. It is necessary to study this behavior in more detail, in order to find better explanations.

The level of c-Met expression varies between C33A and CaSki cells and is inversely related to the expression of miR-23b-3b. Lower levels of c-Met were detected in CaSki cells than in C33A and HaCaT cells. The level of c-Met mRNA was higher in C33A cells. Qian *et al*.^36^ found that silencing HPV16 E6 in CaSki cells restores the expression of p53 and reduces the level of c-Met transcript and protein. Hwang *et al*.^37^ determined that, in SKOV-3 and OSN2 cells, the repression of c-Met results from the binding of p53 to consensus sites in the promoter of the receptor. It is possible that the lower level of c-Met mRNA in CaSki cells than in C33A cells is a product of the binding of residual p53 to the c-Met promoter, although it is also possible that other transcription factors repress the expression of the c-Met gene. On the other hand, the highest level of c-Met mRNA found in C33A cells may be due to the fact that the muted form of p53 in these cells does not inhibit the expression of c-Met. The level of c-Met transcript may be partially regulated by miR-23a-3p, miR-23c, miR-130a-5p (predicted by TargetScan), miR-34a^38^, miR-138^39^, miR-454-3p^40^, and miR-23b-3p. The difference in the expression of c-Met among C33A, CaSki and HaCaT could be explained by the abundance and activity of different miRNAs and other regulatory mechanisms of gene expression.

The overexpression of miR-23b-3p significantly reduces the expression of mRNA and c-Met protein in C33A and CaSki cells, although this effect is not observed in HaCaT cells. These findings concur with that reported by Au-Yeung *et al*.^20^, who showed that the ectopic expression of miR-23b-3p reduces c-Met expression at an mRNA and protein level in SiHa cells. These results, added to those obtained via reporter assays, confirm c-Met as a direct target of miR-23b-3p in C33A and CaSki cells.

The overexpression of miR-23b-3p is significantly related to the phosphorylation of the adaptor protein Gab1 and the kinase Fak in CaSki cells. These results suggest that miR-23b-3p deregulates the processes of proliferation, migration and invasion in CaSki cells via the inhibition of the signals activated by c-Met. It is probable that part of the metastatic potential of CaSki cells is related to the activity of the c-Met/Gab1/FAK pathway. In C33A cells, c-Met may activate different signaling pathways on the c-Met/Gab1/FAK axis. The results suggest that the effect of the overexpression of miR-23b-3p depends on the intrinsic characteristics of the cells.

Previously, we have found that miR-23b-3p levels are reduced in CC tissue^12^. The present study found that the expression of c-Met mRNA is greater in CC tissue than in cervical scraping samples taken from non-SIL patients and that the level of c-Met protein is heterogeneous in CC tissues. The differences in the level of c-Met mRNA in CC tissues and non-SIL cells may be partially due to the tumor suppressor function of miR-23b-3p. The variations in the level of c-Met protein in CC tissues may result from interindividual differences, the specific period of the clinical course in each case, the stage of the tumor and the distinct mechanisms of c-Met regulation, such as the paracrine regulation conducted by the HGF ligand. The heterogenous expression of c-Met in patients may also be the product of the activity of the transcription factors that modulate the expression of c-Met, epigenetic regulation at a chromatin level, and modulation via miRNAs. The mutations or SNPs in the c-Met gene may also alter its expression. Moreover, if the E6 oncoprotein of HPV16 regulates the overexpression of c-Met, it is probable that the viral load and integration HPV16 DNA into the cell genome are factors modulating the expression of c-Met in patients.

## Conclusions

In conclusion, the findings of the present research indicate that miR-23b-3p is a tumor suppressor in CC and contributes to the regulation of the proliferation, migration and invasion of C33A and CaSki cells via the direct regulation of c-Met. In CaSki cells, miR-23b-3p influences the signaling pathway activated by c-Met. The regulation of c-Met by miR-23b-3p is enabled by the binding of miRNA to three MRE sequences located in the 3′-UTR region of c-Met mRNA. These c-Met:miR23b-3p interactions induce a reduction in the mRNA and protein levels of this receptor in C33A and CaSki cells. These results show that, in CC, some biological mechanisms characteristic of cancer are post-transcriptionally regulated by the modulation of the oncogene c-Met and, moreover, show the participation of miR-23b-3p in the regulation of the progression of CC.

## Material and Methods

### Patient samples

The present research studied 13 RNA and 6 protein samples, obtained from the tissue of patients with a cytopathological diagnosis and a negative histological diagnosis for either squamous intraepithelial lesion (SIL) or CC with HPV16 infection. The patients were recruited at the Guerrero State Cancer Institute, located in Acapulco, Guerrero, Mexico. Of these samples, ten RNAs and six proteins corresponded to CC with HPV16, while three RNAs corresponded to non-SIL tissue. All patients signed a letter of informed consent in order to participate in the study. The research was approved by the Research and Ethics Committee at the Guerrero State Cancer Institute and undertaken in accordance with the ethical guidelines set out in the Declaration of Helsinki, made at the World Medical Association General Assembly of 2013.

### Cell lines and culture conditions

The research was conducted on the cell lines for CC, CaSki and C33A. CaSki cells correspond to a metastatic focus of epidermoid cervical carcinoma, located in the small intestine, and contain 600 copies of HPV16 DNA, which are integrated into its genome. This viral genotype is of high oncogenic risk and is more prevalent in CC cases, while C33A cells correspond to cervical carcinoma, are negative to HPV and highly proliferative^41^, and have both metastastic and invasive capacities^42,43^. The cell line HaCaT was included as a normal control for the system. These cells are foreskin keratinocytes, are similar to the basal layer of the keratinocytes of the epidermis, may generate skin equivalents (which resemble the cervical epithelium), and have a high proliferation and differentiation capacity^44,45^. All the cells were obtained from the American Type Culture Collection (ATCC). The CC cells were cultivated in DMEM medium (Invitrogen, Carlsbad, CA. USA), while the HaCaT cells were cultivated in DMEM-F12 medium (Invitrogen, Carlsbad, CA. USA), both supplemented with 10% fetal bovine serum (FBS), 100 U/mL penicillin/100 μg/mL streptomycin, 2 mM L-glutamine and 250 ng/mL fungizone. The cells were kept at 37 °C in a humid atmosphere with 5% CO_2_.

### miRNA expression plasmids for miR-23b-3p

The microRNA expression vector pSilencer 1.0-U6 (Applied Biosystems, Foster, CA. USA) was used to induce the overexpression of miR-23b-3p. This vector contains the RNA U6 promoter, which is recognized by Pol-III, an enzyme that transcribes small RNAs. A DNA insert, which codes for the mature sequence of miR-23b-3p [miRBase: MIMAT0000418], was cloned in the pSilencer 1.0-U6 vector, in order to obtain the pMIR23B-3p plasmid. The sequences for the oligonucleotides used to generate the DNA insert are shown in Table 1. In order to discount the presence of additional restriction enzyme sites, the specificity of the oligonucleotides was confirmed via cross-referencing with existing sequences in GenBank^TM^, using the Nucleotide-BLAST and NEBcutter 2.0 (New England Bio Labs Inc. Ipswich, M. USA) software packages. The alignment of the oligonucleotides in order to generate the DNA insert was conducted in an alignment buffer composed of 30 mM HEPES at pH 7.4, 100 mM potassium acetate, 2 mM magnesium acetate and 4% dimethylsulfoxide (DMSO). The reaction was incubated at 95 °C for 5 min and then at 37 °C for 1 h. The DNA insert was cloned between the restriction sites for *ApaI* and *Eco RI* in the pSilencer 1.0-U6 vector. The plasmid pMIR23B-3p was obtained using the PureYield™ Plasmid Midiprep System (Promega, Madison, WI. USA). The integrity of the pMIR23B-3p plasmid was verified via DNA sequencing in a 3500xl Genetic Analyzer (Applied Biosystems, Foster, CA. USA).

**Table 1:** Oligonucleotides used in this study.

Assay type	Oligonucleotide names and sequences	
**Generation of pMIR23B-3p plasmid**	MIR23-3PF (sense): 5′-ATC ACA TTG CCA GGG ATT ACC TTT TTT-3′ MIR23-3PR (antisense): 5′-AAT TAA AAA AGG TAA TCC CTG GCA ATG TGA TGG CC-3′	
**Generation of reporter plasmids**	pMRE23cMETLuc1 plasmid	pMRE23cMET557F (sense): 5′-CTA GAC TAG TCC AGG GCT GTA GTG CAG TGG TGAT CAT AGA AGC TTG TG-3′ pMRE23cMET577R (antisense): 5′-CAC AAG CTT CTA TGA TCA CAC CAC TGC ACT ACA GCC CTG GAC TAG TCT AG-3′.
	pMRE23cMETLuc2 plasmid	pMRE23cMET1004F (sense): 5′-CTA GAC TAG TGA TGC TAC TCT GAT CTA ATG AAT GTG AAC ATG TAA GCT TGT G-3′. pMRE23cMET1027R (antisense): 5′-CAC AAG CTT ACA TGT TCA CAT TCA TTA GAT CAG AGT AGC ATC ACT AGT CTA G-3′.
	pMRE23cMETLuc3 plasmid	pMRE23cMET2043F (sense): 5′-CTA GAC TAG TTT GTA TAT ACA TTC TTG AGA ACA CTG CAA TGT GAA AAT CAA AGC TTG TG-3′. pMRE23cMET2072R (antisense): 5′-CAC AAG CTT TGA TTT TCA CAT TGC AGT GTT CTC AAG AAT GTA TAT ACA AAC TAG TCT AG-3′.
**RT-qPCR**	c-Met-F (sense): 5′-TAT TTC CCA GAT CAT CCA TTG CA-3′ c-Met-R (antisense): 5′-AAT GTA GGA CTG GTC CGT CAA AA-3′.	
	GAPDH-F (sense): 5′-GGT GAA GGT CGG TGT GAA CG-3′ GAPDH-R (antisense): 5′-CTC GCT CCT GGA AGA TGG TG-3′.	

### Transfection assays with the miRNA expression plasmid

To achieve the overexpression of miR-23b-3p, the cells were transiently transfected with the plasmid pMIR23B-3p, while control cells were transiently transfected with the plasmid pSilencer 1.0-U6 (without DNA insert). The transfection was undertaken using FuGENE HD (Promega, Madison, WI. USA), following the manufacturer’s instructions. Forty-eight hours prior to transfection, 4×10^4^ cells/well were seeded in 24-well plates containing 500 μL of DMEM with 10% FBS and penicillin/streptomycin. At the point of carrying out the transfection, the plasmids and FuGENE were diluted in FBS-free medium and incubated for 15 min at room temperature. All the assays were undertaken with 5 μg for each plasmid. The cells were incubated with plasmid/FuGENE complex for 4–6 h, rinsed and the medium then replaced with DMEM or DMEM-F12 supplemented with 10% FBS. After 48 h of transfection, the cells were harvested and used to obtain the total RNA and the proteins which were used for the real time RT-qPCR and Western Blot assays, respectively. The transfection assays were repeated, independently, at least three times.

### Real-time quantitative polymerase chain reaction (RT-qPCR)

The total RNA of the transfected and non-transfected (N/T) cells was obtained using TriPure reagent (Roche, Basel, Switzerland). The expression of miR-23b-3p was measured via RT-qPCR using TaqMan assays for microRNAs, following the manufacturer’s instructions. The expression of miR-92a was used as a reference control for normalizing the expression of miR-23b-3p. The Ct values were used to compare the expression of miR-23b-3p in the cells transfected with the pMIR23B-3p and pSilencer 1.0-U6 plasmids and the N/T cells. The relative expression was calculated using the 2^−ΔCt^ method. The RT-qPCR assays were conducted in triplicate.

The expression of c-Met mRNA was determined via RT-qPCR before and after the cells were transfected. The measurement was carried out in the real time BioRad C1000/CFX96 system, using the One-Step SYBR® PrimerScripTM RT-PCT kit II (Takara, Clontech, Japan), in accordance with the manufacturer’s instructions. Each RT-qPCR reaction used 200 ng total RNA and specific oligonucleotides for c-Met. The housekeeping gene glyceraldehyde 3-phosphate dehydrogenase (GAPDH) was used as an endogenous control. The oligonucleotide sequences are described in Table 1. The amplification program consisted of the following: an initial stage at 42 °C for 5 min for the synthesis of cDNA; an RT inactivation stage at 95 °C for 5 min, and 40 cycles applied at 95 °C for 3 s, at 60 °C for 30 s and at 72 °C for 30 s; and, a dissociation stage at 72 °C for 2 min. The changes in the expression of c-Met were calculated using the Ct comparative method. Each assay was repeated three times, independently.

### Western blot assays

Forty-eight hours after the transfection, the cells were harvested and used as a protein source for the Western Blot assays. The proteins were obtained using TriPure reagent (Roche, Basel, Switzerland), precipitated all night with absolute acetone based on the organic phase of TriPure and centrifuged at 12,000 rpm and 4 °C for 10 min. The precipitates were washed three times with guanidine hydrochloride [0.3 M] diluted in ethanol to 95%, homogenized, centrifuged at 9000 rpm for 7 min at 4 °C, and incubated for 20 min at room temperature. The proteins were washed with absolute ethanol, dried in an extraction hood, diluted in SDS to 4% and quantified using the Pierce® BCA protein assay kit (Pierce, Rockford, IL. USA). Thirty micrograms of total proteins were separated via SDS-PAGE to 10% and transferred to a 0.45 μm nitrocellulose membrane in a wet system. A pre-dyed and biotinylated molecular weight marker was included, and GAPDH used as a load control. The membranes were blocked with 5% low fat milk, diluted in Tris-buffered saline with Tween 20 (TBST 1×) for 1–2 h at room temperature and constant agitation, washed three times with TBST 1X and incubated all night at 4 °C with the corresponding primary antibody (Table 2). The membranes were incubated with secondary antibodies coupled to HRP (Table 2). The detection of proteins was carried out via potentiated chemiluminescence, using the Clarity^TM^ Western ECL substrate system (170–5060, Bio-Rad, Germany), in ChemiDoc^TM^ MP Imaging equipment (Bio-Rad, Germany). The relative expression of proteins was analyzed and represented as the radius of density of the problem protein**/**the density of the GAPDH protein. Each assay was repeated three times, independently.

**Table 2:** Antibodies used in the study.

Primary antibodies	Catalog number/distributor	Dilution	Secondary antibodies (Catalog number/distributor)	
			Anti-rabbit IgG-HRP 7075P2/CST, EE. UU.	Anti-mouse IgG-HRP 115-035-003/JIR, EE. UU.
Anti-c-Met	D1C2/CST EE. UU.	1:1000	1:3000	—
Anti-Gab1	3232/CST, EE. UU.	1:1000	1:5000	—
Anti-phospho Gab1(Tyr-307)	3234/CST, EE. UU.	1:3000	1:5000	—
Anti-Fak	D2R2E-13009/CST, EE. UU.	1:3000	1:5000	—
Anti-phospho Fak (Tyr-397)	D20B1-8556/CST, EE. UU.	1:1000	1:5000	—
Anti-GAPDH	(6C5)-sc-32233/SCBT, EE. UU.	1:1000	—	1:20,000

CST: Cell Signaling Technology; SCBT: Santa Cruz Biotechnology; JIR: Jackson ImmunoResearch.

### Plasmid reporter and luciferase activity assays

The cells were transiently transfected with the reporter plasmids pMRE23cMETLuc1, pMRE23cMETLuc2 and pMRE23cMETLuc3, which contain the cloned sequences MRE23-1 (6mer), MRE23-2 (8mer) and MRE23-4 (8mer), respectively. The microRNA response elements (MRE) for miR-23b-3p analyzed in the present study were located in positions 4730 to 4750, 5177 to 5200 and 6216 to 6245 from the 3′-UTR region of the c-Met mRNA. The *in silico* analysis of the MRE23 was supported by the NCBI Reference Sequence Database of search nucleotide sequences for both the humane gene c-Met (NCBI: NM_000245.3) and miR-23b-3p (NCBI: NR_029664.1 and miRBase: MIMAT0000418). The prediction of MRE23 sites was made on the bioinformatic platforms TargetScan (http://www.targetscan.org/vert_71/), miRTarBase (http://mirtarbase.mbc.nctu.edu.tw/php/index.php), microRNA.org (http://34.236.212.39/microrna/home.do), miRDB (http://mirdb.org/), RNA22 (https://cm.jefferson.edu/rna22/) and PicTar (https://pictar.mdc-berlin.de/). Each MRE23 site was included in an oligonucleotide sequence, which was hybridized with a complementary oligonucleotide in order to generate DNA inserts, using an alignment buffer (30 mM HEPES pH 7.4, 100 mM potassium acetate, and 2 mM magnesium acetate). Each alignment reaction was incubated at 95 °C for 10 min, after which, the temperature was gradually reduced for an hour. The oligonucleotide sequences used for the generation of all the DNA inserts are described in Table 1.

The DNA inserts were individually cloned between the restriction sites for *Spe I* and *Hind III* in the pMIR-Report- Luciferase reporter vector (Life Technologies, Carlsbad, CA. USA), which contains the reporter gene for luciferase downstream of the CMV promoter/termination system. All the plasmids were purified using the PureYield Plasmid Midiprep System (Promega, Madison, WI. USA), while the fidelity of their sequences was verified via DNA sequencing.

The co-transfection assays were conducted using the plasmid pMIR23B-3p in order to induce the expression of miR-23b-3p. All the transfection and co-transfection assays were carried out with 1 μg of each plasmid, using the FuGENE HD reagent (Promega, Madison, WI, USA). A total of 4 × 10^4^ cells/well were seeded in 24-well plates and incubated at 37 °C, 24 h prior to transfection. The cells were transfected with the above described plasmids and incubated for 4–6 h in the absence of FBS.

The culture medium was replaced with supplemented medium, and the cultures were incubated under the same conditions for 48 h. The cells were washed twice with PBS 1X pH 7.4, harvested and treated with lysis buffer (20 mM Tris-HCl pH 7.4, 10 mM NaCl, 10 mM KCl, 3 mM MgCl_2_, 0.5% Triton X-100, 0.5% Nonidet P40). The lysate products were centrifuged at 12,000 rpm for 10 min at 4 °C, in order obtain the proteins from the supernatant. Fifty micrograms of the total proteins were used to determine luciferase activity using the Dual-Glo luciferase assay kit (Promega, Madison, WI, USA) in a Glomax multi-detection system (Promega, Madison, WI, USA). The luminescence was normalized in relation to the plasmid pMIR-Report-Luciferase, without a DNA insert. All the determinations were repeated over three different times, independently.

### Cellular proliferation assays

Cellular proliferation was measured using the CellTiter 96 AQueous Non-Radioactive Cell Proliferation Assay (MTS) kit (Promega, Madison, WI, USA). A total of 0.5×10^4^ cells/well (transfected with the plasmid pMIR23B-3p or the plasmid pSilencer 1.0-U6, –without DNA insert– and N/T cells) were seeded in 96-well plates and incubated at 37 °C. After 24 h of incubation, 20 µL of the MTS reagent, and 100 µL of fresh medium supplemented with 5% FBS were added to each well, ensuring that the plate was not exposed to any type of light source. The plates were incubated at 37 °C for 4 h. Tetrazolium salts, components of the reagent MTS, were bio-reduced by the live cells into a colored formazan product soluble in the culture medium. Absorbency in the medium of transfected cells and NT was measured at 490 nm, in a Glomax multi-detection system (Promega, Madison, WI, USA). The absorbency readings were taken every 24 h until 96 h of transfection had been achieved. The cellular proliferation was calculated with the formula: % proliferation = (average experimental absorbance/average control absorbance) × 100. The N/T cells were used as a negative control for transfection. Every assay was carried out three times, independently.

### Cellular migration assays

The capacity for cellular migration was evaluated via wound healing assay. Cells transfected with 5 µg of the plasmid pMIR23B-3p or 5 µg of the plasmid pSilencer 1.0-U6 (without DNA insert) and N/T cells were cultivated in 6-well plates until reaching a confluence of 100%. With the tip of a 200 μL micropipette, a stria was scraped onto the cellular monolayer, and the cultures were washed twice with PBS 1X in order to remove the displaced or damaged cells and incubated at 37 °C in FBS-free medium for 48 h. The cells that migrated to the center of the wound were documented via microphotographs, 0, 24, and 48 h after the stria had been made. The assays were undertaken in three independent replicates.

### Cellular invasion assays

Cellular invasion was determined in 1 × 10^5^ cells, after 72 h of transfection with 5 µg of the plasmid pMIR23B-3p or the plasmid pSilencer 1.0-U6 (without DNA insert), as well in N/T cells.

The cells were deposited onto a Transwell insert with an 8.0 μm polycarbonate membrane covered with matrigel (Corning®, NY, USA). The cells were seeded in 24-well plates, over the surface of the insert, with FBS-free medium. Medium supplemented with 20% FBS was added below the insert.

The plates were incubated at 37 °C in a 5% CO_2_ atmosphere. The cells remaining on the surface of the insert were removed after 24 h, while the cells that had invaded, passing through the membrane, were fixed with cold 4% paraformaldehyde for 10 min and dyed with 0.1% crystal violet for 15 min. Cellular invasion was documented by microphotographs, and the cells that had invaded were counted in six fields/insert. The assays were carried out in triplicate.

### Statistical analysis

The data analysis used the GraphPad Prism 8.0 software (Inc, San Diego, CA. USA). The histograms represent the values of the averages and the bars indicate the magnitude of the standard error of the averages. A two-tailed student’s t-test was used to compare the value of the averages between two groups. In order to analyze the quantitative non-parametric variables and compare the data for the two groups, the Mann-Whitney U test was used. A value of p < 0.05 was considered statistically significant and was marked with an asterisk (*). The authors of the present study declare that the data fully available.

## Supplementary information

Supplementary information.
