# Supplementary material for: MiR-23b-3p reduces the proliferation, migration and invasion of cervical cancer cell lines via the reduction of c-Met expression
Source: Sci Rep. 2020 Feb 24;10:3256. doi: 10.1038/s41598-020-60143-x (PMC7039958; doi:10.1038/s41598-020-60143-x)
Supplement: Supplementary file 1 — Supplementary information. [file 41598_2020_60143_MOESM1_ESM.pdf]

Title Page

MiR-23b-3p reduces the proliferation, migration and invasion of cervical cancer cell lines via the reduction of c-Met expression

Gabriela Elizabeth Campos-Viguri[1], Oscar Peralta-Zaragoza[2], Hilda Jiménez-Wences[1], Alma Edith Longinos-Gonzalez[1], Carlos Alberto Castañón-Sánchez[3], Miriam Ramírez-Carrillo[4], César López Camarillo[5], Eduardo Castañeda-Saucedo[6], Marco Antonio Jiménez-López[7], Dinorah Nashely Martínez-Carrillo[1], and Gloria Fernández-Tilapa[1*]

[1.] Laboratorio de Investigación Clínica. Facultad de Ciencias Químico-Biológicas, Universidad Autónoma de Guerrero. Avenida Lázaro Cárdenas S/N, Colonia Haciendita, Chilpancingo, Guerrero, México. 39070.

[2.] Dirección de Infecciones Crónicas y Cáncer; Centro de Investigación en Enfermedades Infecciosas. Instituto Nacional de Salud Pública. Av. Universidad No. 655, Cerrada los Pinos y Caminera. Colonia Santa María Ahuacatitlán, Cuernavaca, Morelos, México 62100.

[3.] Hospital Regional de Alta Especialidad, Oaxaca, Oaxaca, México.

[4.] Laboratorio de Química de Productos Naturales, Facultad de Farmacia, Universidad Autónoma del Estado de Morelos. Cuernavaca, Morelos, México.

[5.] Posgrado en Ciencias Genómicas, Universidad Autónoma de la Ciudad de México, CDMX, México.

[6.] Laboratorio de Biología Celular del Cáncer. Facultad de Ciencias Químico-Biológicas, Universidad Autónoma de Guerrero. Chilpancingo, Guerrero, México. 39070.

[7.] Instituto Estatal de Cancerología “Dr. Arturo Beltrán Ortega”, Acapulco, Guerrero, México.

* Corresponding author: Dra. Gloria Fernández-Tilapa
Facultad de Ciencias Químico Biológicas
Universidad Autónoma de Guerrero.
Avenida Lázaro Cárdenas S/N, Colonia Haciendita,
Chilpancingo, Guerrero, México. 39070.
Tel: (+52)-747-47 25503
Email: gferti@hotmail.com

Blots used for membrane construction

c-Met is a direct target of miR-23b-3p in CC cells

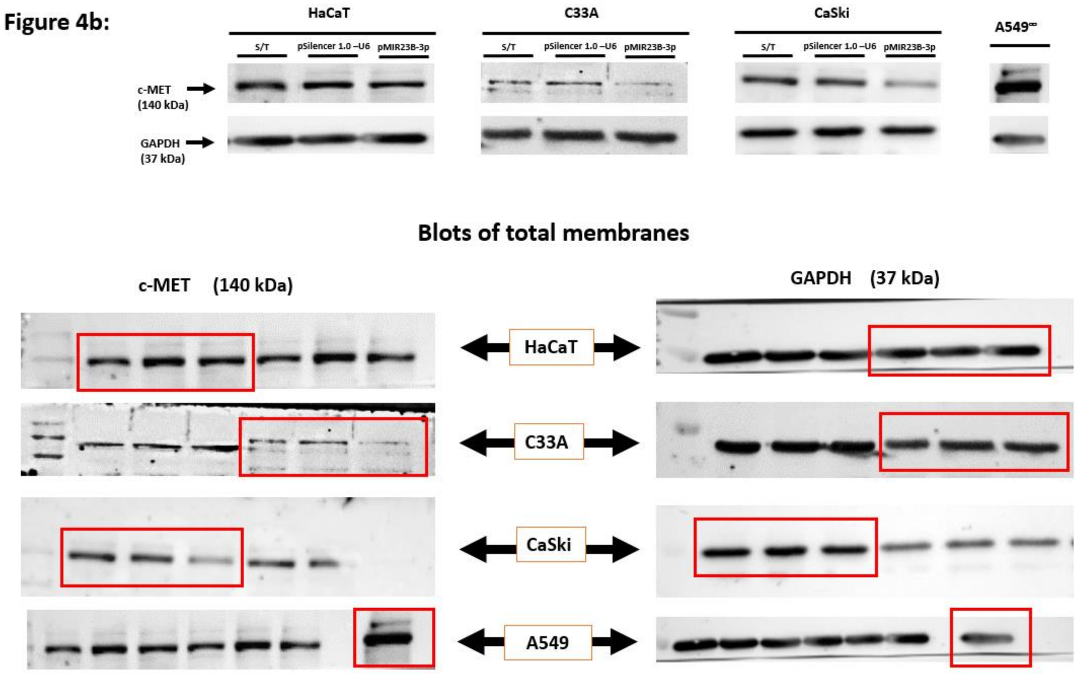

Supplementary figure S1: Complete blots of figure 4b.

miR-23b-3p modifies the activation of Gab1 and Fak in CaSki cells

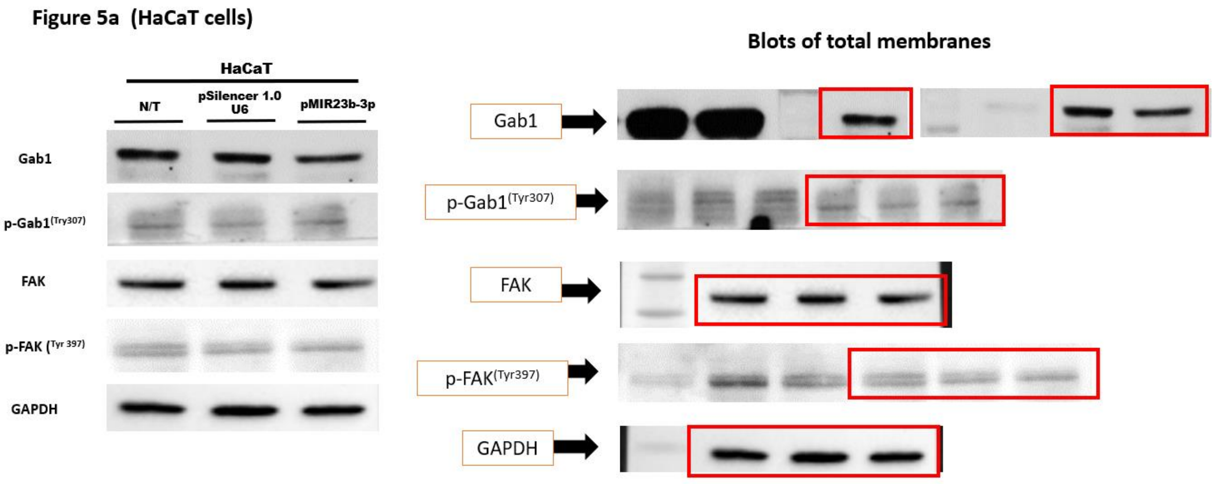

Supplementary figure S2: Complete blots of figure 5a- HaCaT cells.

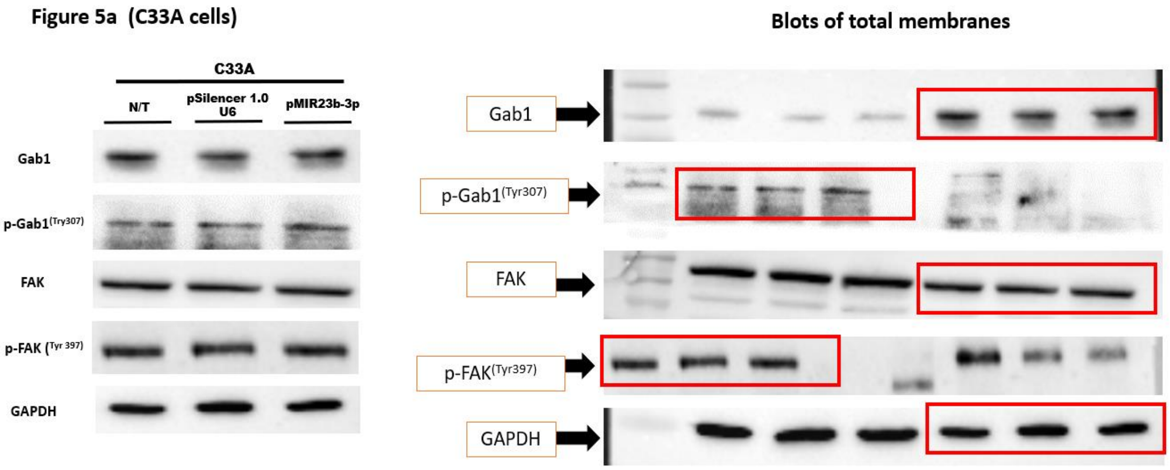

Supplementary figure S3: Complete blots of figure 5a- C33A cells.

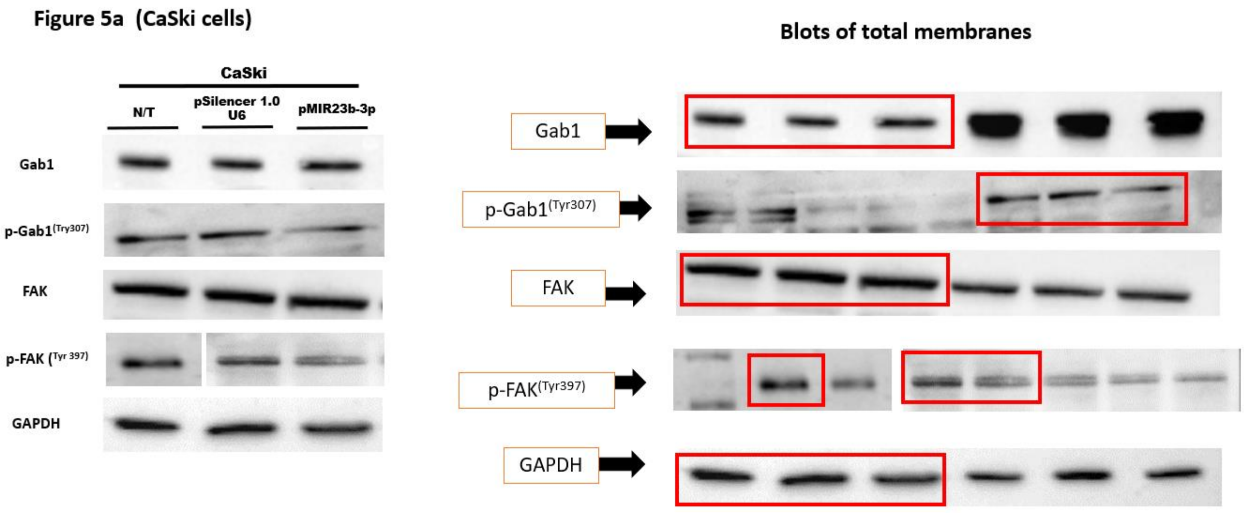

Supplementary figure S4: Complete blots of figure 5a- CaSki cells.

The c-Met protein is expressed heterogenously in HPV16-positive CC patients

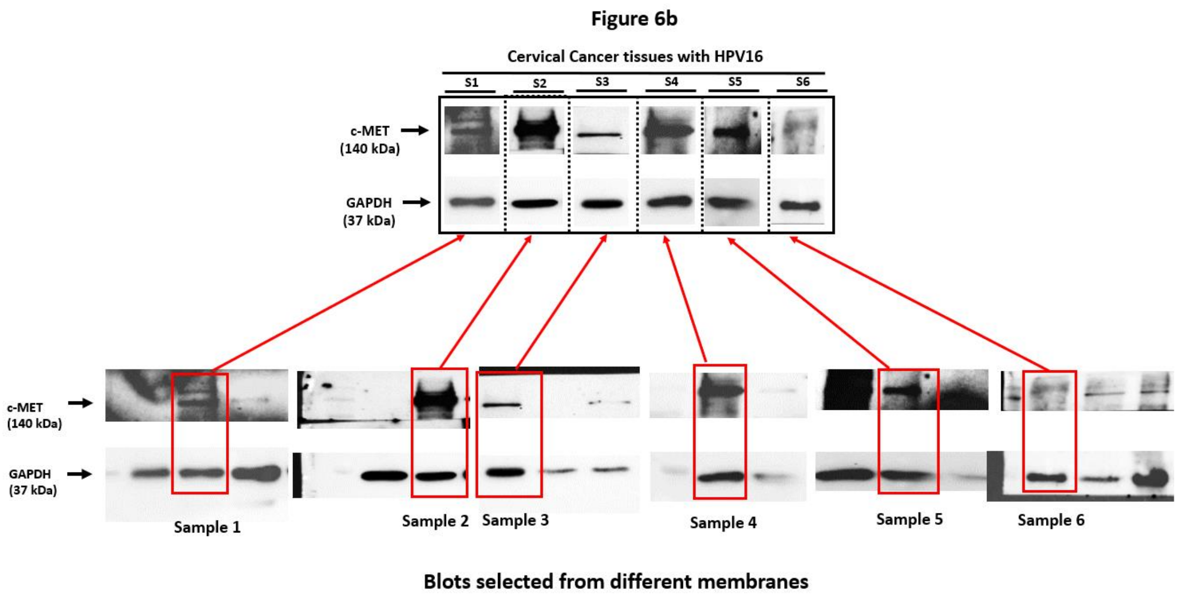

Supplementary figure S5: Complete blots of figure 6b. The image from the Western Blot assay was constructed based on bands located in different positions in the same membrane and independent assays.
